# Daily tadalafil administration improves cardiac autonomic regulation in men with non-organic erectile dysfunction: a prospective heart rate variability study

**DOI:** 10.1093/sexmed/qfag029

**Published:** 2026-05-11

**Authors:** Görkem Yıldız, Tolga Kunak

**Affiliations:** Faculty of Medicine, Department of Cardiology, Yüksek İhtisas University, Ankara 06530, Türkiye; Faculty of Medicine, Department of Cardiology, Akdeniz University, Antalya 07070, Türkiye

**Keywords:** erectile dysfunction, tadalafil, heart rate variability, autonomic nervous system, cardiovascular risk, phosphodiesterase-5 inhibitor

## Abstract

**Background:**

Non-organic ED patients have cardiac autonomic dysfunction measurable by heart rate variability (HRV). Tadalafil reduces cardiovascular mortality, but exact mechanisms remain unclear.

**Aim:**

To evaluate daily tadalafil effects on cardiac autonomic regulation in non-organic ED and identify clinical predictors of HRV response.

**Methods:**

In this prospective study, 82 men with non-organic ED (mean age 46.8 ± 5.0 years) received tadalafil 5-10 mg daily. Assessment included International Index of Erectile Function-Erectile Function (IIEF-EF), echocardiography, laboratory tests, and 24-hour Holter ECG. HRV was analyzed via time-domain (standard deviation of NN intervals [SDNN], root mean square of successive differences [RMSSD]) and frequency-domain (low-frequency power [LF], high-frequency power [HF], LF/HF ratio) parameters. Post-treatment recordings were blindly evaluated. Early response to tadalafil was defined as achievement in the severity-adjusted Minimal Clinically Important Difference (MCID) in IIEF-EF or final IIEF-EF score ≥26.

**Outcomes:**

Primary: ΔSDNN. Secondary: ΔHF power, ΔLF/HF ratio.

**Results:**

IIEF-EF improved significantly (15 to 23, *P* < .001) and 74.4% were early responders (*n* = 61). In the overall cohort, SDNN increased (42.39 to 52.39 ms, *P* < .01), HF power increased (267.86 to 294.44 ms^2^,*P* < .001), and LF/HF ratio decreased (2.78 to 2.31, *P* < .01). Both groups showed HRV improvements without between-group differences (*P* > .05), suggesting autonomic modulation independent of erectile response. Multivariable regression: diabetes negatively predicted SDNN improvement; higher testosterone, higher baseline IIEF-EF score, and lower BMI predicted greater HF enhancement. Only 1.1% of patients discontinued the treatment (due to dyspepsia).

**Clinical Implications:**

Daily tadalafil treatment may enhance parasympathetic modulation in men with non-organic ED regardless of clinical erectile improvement, suggesting a potential mechanism for its observed cardiovascular benefits.

**Strengths and Limitations:**

Strengths include rigorous, blinded Holter assessment. Limitations include the lack of a placebo control, short follow-up (28 days), and a small non-responder subgroup.

**Conclusion:**

Daily tadalafil enhanced parasympathetic modulation and reduced sympathovagal imbalance independently of erectile improvement.

## Introduction

Erectile dysfunction (ED) is the persistent or intermittent inability to achieve or maintain an erection sufficient for satisfactory sexual intercourse.^1^ It is multifactorial and shares major risk factors with cardiovascular disease, including age, diabetes, obesity, dyslipidemia, metabolic syndrome, smoking, and physical inactivity.^2^ Beyond this overlap, ED has been recognized as an independent predictor of cardiovascular events, with risks comparable to those associated with smoking or family history of myocardial infarction.^3^ Meta-analyses have shown that men with ED have higher rates of cardiovascular events, myocardial infarction, cerebrovascular events, and all-cause mortality than men without ED.^4^ This association is particularly pronounced in younger men, supporting the view that ED may represent an early manifestation of systemic endothelial dysfunction and subclinical atherosclerosis.^5^

Autonomic imbalance also appears to contribute to ED pathophysiology. Penile erection is predominantly mediated by parasympathetic activation, whereas sympathetic activity predominates during detumescence.^6^ Cardiac autonomic regulation can be assessed noninvasively using heart rate variability (HRV),^7^ and several studies have demonstrated sympathetic predominance and parasympathetic withdrawal in men with non-organic ED.^8–12^ These findings suggest that autonomic dysfunction may be one of the mechanisms linking ED and cardiovascular risk.^13^

Phosphodiesterase-5 inhibitors (PDE-5Is) are recommended first-line therapy for ED.^14,15^ By enhancing nitric oxide–cGMP signaling, these agents improve vasodilation in penile and systemic vascular beds.^16^ Observational studies have suggested that PDE-5I use may be associated with lower cardiovascular event rates and mortality in men with ED,^17,18^ although causality and the biological basis of these associations remain uncertain.

Because HRV is a well-established surrogate of autonomic function and cardiovascular risk,^7,19^ we hypothesized that tadalafil might improve cardiac autonomic balance in men with non-organic ED. Therefore, this prospective observational study evaluated changes in HRV after approximately 30 days of daily tadalafil therapy, with secondary aims of identifying predictors of HRV improvement and exploring whether autonomic changes differed according to early erectile-function response. Specifically, we asked: does 30-day daily tadalafil administration significantly improve cardiac autonomic regulation—as quantified by SDNN, HF power, LF power, and the LF/HF ratio—in men with non-organic ED, and does the magnitude of this autonomic improvement differ between patients who achieve an early erectile function response and those who do not?

## Methods

This was a prospective, single-arm, observational cohort study conducted at the Outpatient Clinic of Yüksek İhtisas University, between August 15, 2024, and February 15, 2025. The study protocol was approved by the local ethics committee (Ref:2024/05/18, dated July 31, 2024), and all participants provided written informed consent.

To reduce bias, we used consecutive sampling with predefined criteria, blinded urological and cardiological assessments, standardized Holter protocols with quality control, and within-subject plus multivariable analyses. However, the open-label single-arm design still limits control of performance and expectation bias.

Prior to treatment, 122 patients underwent a detailed physical examination, anthropometric measurements, International Index of Erectile Function-Erectile Function domain (IIEF-EF), transthoracic echocardiography (Affiniti CVx or EPIC S4-1 probe, Phillips Healthcare Systems, Andover, MA, United States), laboratory tests (total cholesterol, low-density lipoprotein, high-density lipoprotein, creatinine, hemoglobin, and thyroid function tests, total and free testosterone level), and 24-hour Holter electrocardiogram (ECG) monitoring (BTL-08, Holter H600, BTL Industries Ltd., Hertfordshire, United Kingdom) by urologist and cardiologist. As a result of these evaluations, a total of 26 patients with diagnoses of coronary artery disease (*n =* 4), peripheral artery disease (*n =* 2), atrial fibrillation (*n =* 4), and heart failure (*n =* 2) were excluded from the study together with those using antiarrhythmic drugs (*n =* 14).

### 24-hour Holter ECG monitoring

The 24-hour Holter ECG monitoring (BTL-08, Holter H600, BTL Industries Ltd., Hertfordshire, United Kingdom) was initiated between 8:00 AM and 8:30 AM for all patients to minimize circadian variability, and recordings were terminated after 24 hours. During monitoring, patients were instructed to continue their daily routine and to keep an activity dairy. Using the activity dairy, sleep and wake times were delineated to standardize the influence of circadian rhythm. Patients were asked to maintain consistent daily consumption of caffeine/theine and, when applicable, nicotine (electronic or conventional cigarettes) during both monitoring periods, and potential differences between the 2 assessments were subsequently evaluated. Analysis of the 24-hour ECG recordings was performed using CardioPoint–Holter H600 software (BTL Industries Ltd., Hertfordshire, United Kingdom). Prior to HRV analysis, recordings with interference were automatically edited by the software and manually reviewed/edited by a cardiologist when necessary. If the initial Holter recording had an artifact burden >10% (eg, noise, signal loss, or annotation errors), monitoring was repeated; if the repeated recording still showed an artifact burden >5%, the recording was excluded from HRV analyses (*n =* 8). HRV analysis was performed according to the standards established by the Task Force of the European Society of Cardiology and the North American Society of Pacing and Electrophysiology.^20,21^ Time-domain parameters included SDNN and RMSSD, calculated from the entire 24-hour recording. Frequency-domain analysis was derived from the 24-hour recording using Fast Fourier Transform (FFT) and included LF (0.04–0.15 Hz), HF (0.15–0.40 Hz), and the LF/HF ratio. The post-treatment 24-hour ECG recordings were analyzed by a different cardiologist who was unaware of the baseline and follow-up results.

### Tadalafil treatment and follow-up

This study enrolled patients diagnosed with non-organic ED by a urologist following a comprehensive evaluation that included medical history, physical examination, laboratory tests, and the IIEF-EF domain score. These patients were initiated on a standard dose of 5 mg/day tadalafil.

Patients with non-organic ED were started on tadalafil 5 mg/day after comprehensive urological evaluation. Those with suspected organic ED underwent additional testing according to routine practice and AUA guidance and were not enrolled if an organic cause was identified.^14^ Patients were re-evaluated at weeks 2 and 4 for efficacy and adverse effects. At week 2, dose escalation to 10 mg/day was performed in patients who failed to achieve the severity-adjusted Minimal Clinically Important Difference (MCID) in IIEF-EF or whose total IIEF-EF remained <26. At week 4, patients were classified as early responders if they achieved either the severity-adjusted MCID (≥2 points for mild ED, ≥5 points for moderate ED, and ≥ 7 points for severe ED) or a final IIEF-EF score ≥ 26; the remainder were classified as non-early responders.^22^ Because this assessment occurred earlier than the 12-week period generally used to define treatment non-response, these groups were labeled early responders and non-early responders rather than definitive responders and non-responders.^14^

**Image 1:**
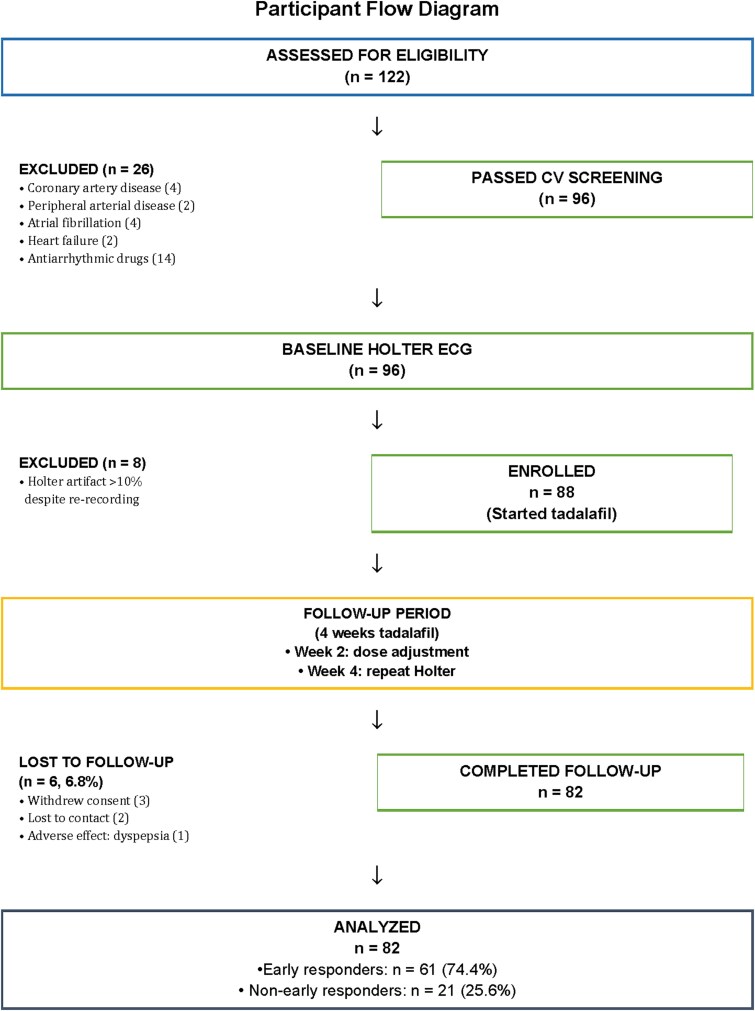
Participant flow diagram. Flow diagram showing participant enrollment and attrition. Of 122 screened, 26 were excluded for cardiovascular comorbidities or antiarrhythmic use, 8 for poor Holter quality (artifact >10% despite re-recording), and 6 were lost to follow-up (3 withdrew, 2 lost contact, 1 dyspepsia). Final analysis: 82 men with non-organic erectile dysfunction who completed tadalafil therapy and both Holter assessments.CV, cardiovascular; ED, erectile dysfunction; ECG, electrocardiography; HRV, heart rate variability.

A total of 122 consecutive patients were screened; 26 were excluded for cardiovascular comorbidities or antiarrhythmic use, and 8 for poor Holter quality despite re-recording. Of 88 enrolled patients, 6 (6.8%) were lost to follow-up (3 withdrew, 2 lost contacts, 1 dyspepsia). The final cohort comprised 82 men who completed tadalafil therapy and both Holter assessments (Image 1).

### Statistical analysis

IBM SPSS Statistics 25.0 (IBM Corp., Armonk, NY, United States) was used for all analyses. The normality of continuous variables was assessed using the Shapiro–Wilk test. Normally distributed data are presented as mean ± SD, whereas non-normally distributed data are presented as median (IQR).

Within-group pre–post comparisons were performed using the paired Student’s *t* test for normally distributed variables and the Wilcoxon signed-rank test for non-normally distributed variables. Categorical variables were compared using the chi-square test.

For variables showing a significant pre–post change, within-subject change scores (Δ) were calculated. Associations between change scores and independent variables were evaluated using multiple linear regression. Because LF, HF, LF/HF, ΔSDNN, and ΔRMSSD were non-normally distributed, log-transformation was attempted; however, normality was not achieved after transformation. Accordingly, non-parametric methods were retained for inferential testing of these parameters, and regression models were interpreted with caution. Analysis of covariance (ANCOVA) was used to control for baseline values when evaluating predictors of post-treatment outcomes. Subgroup analyses compared tadalafil early responders and non-early responders using change scores (Δ). Potential confounders evaluated in multivariable models included baseline HRV parameters, age, body mass index, comorbidities (diabetes mellitus, hypertension, dyslipidemia), smoking status, total testosterone level, and change in erectile function (ΔIIEF-EF). Multicollinearity was assessed by inspecting variance inflation factors (VIF <5 for all variables).

### Endpoints and multiplicity control

SDNN was pre-specified as the primary HRV endpoint because time-domain indices derived from 24-hour Holter recordings demonstrate higher test–retest stability than frequency-domain metrics. HF power and the LF/HF ratio were pre-specified as secondary HRV endpoints. To control the family-wise type I error across HRV endpoints, we applied a hierarchical testing strategy (gatekeeping) in the following order: (1) ΔSDNN (primary), (2) ΔHF (secondary), and (3) ΔLF/HF (secondary). Secondary HRV endpoints were considered statistically supported only if the preceding endpoint in the hierarchy reached statistical significance in the overall cohort. All early responder/non-early responder subgroup analyses were considered exploratory.

### Missing data and sensitivity analysis

Six patients (6.8%) were lost to follow-up (3 withdrew, 2 lost contact, 1 discontinued due to dyspepsia). Complete-case analysis was performed (*n =* 82, no imputation). Sensitivity analysis assuming worst-case scenario (dropouts Δ = 0, *n =* 88) confirmed robust results: ΔSDNN +7.5 ms (*P* = .027) versus +8.0 ms (*P* = .021); ΔHF +168 ms^2^ (*P* = .031) versus +185 ms^2^ (*P* = .018). Attrition did not materially affect conclusions.

## Results

### Baseline characteristics

Of 122 screened, 82 men completed the study protocol after excluding 26 for cardiovascular contraindications, 8 for Holter quality issues, and 6 lost to follow-up (Image 1). All 82 participants met HRV quality criteria; 5 required repeat Holter acquisition. Baseline characteristics are summarized in Table 1. The mean age of the cohort was 46.8 ± 5.0 years, with high burden of cardiometabolic risk factors and a mean 10-year cardiovascular event risk was 5.5 ± 2.8%.The average duration of tadalafil use was 29.6 ± 2.9 days (total dose 180.9 ± 46.5 mg). Median baseline IIEF-EF was 15 (11.25), and 61 patients (74.4%) were classified as early responders and 21 (25.6%) as non-early responders (Table 1).

**Table 1:** General clinical characteristics.

Age, years, mean ± SD	46.8 ± 5.0
Smoking, % (n)	43.9% (36)
Diabetes mellitus, % (n)	24% (20)
Hypertension, % (n)	22% (18)
Family history of premature CAD, % (n)	20.7% (17)
Body mass index, kg/m^2^, mean ± SD	25.8 ± 1.5
Systolic blood pressure, mmHg, mean ± SD	125.6 ± 3.3
Diastolic blood pressure, mmHg, mean ± SD	82.2 ± 3.8
Fasting blood glucose, mg/dL, mean ± SD	97.9 ± 3.9
Creatinine, mg/dL, median (IQR)	1.11 (0.2)
Total cholesterol, mg/dL, mean ± SD	185.2 ± 6.5
LDL, mg/dL, mean ± SD	145.8 ± 8.7
HDL, mg/dL, mean ± SD	33.6 ± 3.4
Triglycerides, mg/dL, mean ± SD	194.5 ± 13.3
Hemoglobin, g/dL, mean ± SD	13.2 ± 0.5
TSH, mg/dL, mean ± SD	3.17 ± 0.25
Total testosterone, mU/L, median) (IQR)	419 (50.5)
Free testosterone, ng/dL, mean ± SD	12.07 ± 2.72
Total tadalafil dose^*^, mg, mean ± SD	180.9 ± 46.5
Treatment duration, days, mean ± SD	29.6 ± 2.9
SCORE 2, %, mean ± SD	5.5 ± 2.8%
IIEF-EF Score	
Baseline, median (IQR)	15 (11.25)
2^nd^ week, median (IQR)	21 (13.25)
4^th^ week, median (IQR)	23 (12.0)

^*^Patients escalated to 10 mg, *n* (%): overall: 45.1(%); early responders: 26.2 (%); non-early responders:100 (%).

CAD, coronary artery disease; IIEF-EF, International Index of Erectile Function-Erectile Function domain; HDL, high-density lipoprotein; LDL, low-density lipoprotein; SCORE 2, Systematic COronary Risk Evaluation 2; TSH, thyroid-stimulating hormone.

### Erectile function response

IIEF-EF scores improved significantly in the overall cohort and in both responder strata (all *P* < .001, Table 2). In the overall cohort (*n =* 82), the median post-treatment IIEF-EF was 23 (IQR:12.0). Based on the predefined response criteria (ΔIIEF-EF ≥4 or final IIEF-EF ≥ 26), 61 patients (74.4%) were classified as early responders and 21 (25.6%) as non-early responders. Between-group comparison of IIEF-EF change shows a statistically significant difference (Mann–Whitney U: U = 8.5, Z = −6731, *P* < .001).

**Table 2:** Pretreatment and posttreatment 24-hour Holter ECG parameters and IIEF score in tadalafil early responders and non-early responders’ subgroup.

Overall (*n* = 82)				
	Pretreatment	Post-treatment	Delta (∆)	*P*
IIEF-EF score	15.0 (11.25)	23.0 (12.0)	6.0 (7.0)	<.001
SDNN (ms)	42.39 (14.40)	52.39 (8.06)	7.26 (9.93)	<.01
RMSSD (ms)	26.60 (10.48)	29.58 ± 2.73	3.24 (13.11)	.07
LF (ms^2^)	607.04 (651.80)	655.87 (637.08)	0.0 (0.2)	.493
HF (ms^2^)	267.86 (241.56)	294.44 (232.33)	25.97(84.56)	<.001
LF/HF ratio	2.78 (1.89)	2.31 (1.47)	−0.18 (1.01)	<.001
Early responders subgroup (*n* = 61)				
IIEF-EF score	16.0 (10.0)	26.0 (7.5)	9.0 (6.0)	<.001
SDNN (ms)	42.38 (17.06)	52.20 (7.11)	7.26 (11.70)	<.001
RMSSD (ms)	26.55 (9.91)	30.11 (3.83)	3.24 (11.36)	.108
LF (ms^2^)	654.43 (733.17)	689.27(640.44)	0.0 (0.01)	.565
HF (ms^2^)	324.72 (243.45)	389.55 (209.67)	19.04 (80.61)	.003
LF/HF ratio	2.63 (2.01)	2.26 (1.49)	−0.13 (0.61)	.001
Non-early responders subgroup (*n* = 21)				
IIEF-EF score	12.0 (9.5)	14.0 (10.0)	2.0 (2.0)	<.001
SDNN (ms)	43.14 (16.42)	53.66 (10.80)	9.08 (8.62)	<.001
RMSSD (ms)	27.07 (13.51)	29.22 (3.89)	1.77 (15.46)	.322
LF (ms^2^)	556.84 (289.13)	556.84 (299.31)	0 (0.01)	.329
HF (ms^2^)	155.3 (86.34)	198.44 (106.17)	44.79 (80.94)	.001
LF/HF ratio	3.09 (3.09)	2.81 (1.03)	−0,60 (1.96)	.003

^*^Patients escalated to 10 mg, *n* (%): overall: 45.1(%); early responders: 26.2 (%); non-early responders:100 (%). Parameters with normal distribution were presented as mean ± SD, while parameters without normal distribution were presented as median (IQR). Δ values are median of within-subject differences. HF = high-frequency power; IIEF = International Index of Erectile Function; LF, low-frequency power; LF/HF = low to high frequency ratio; ms, millisecond; RMSSD, root mean square of successive differences; SDNN, standard deviation of NN intervals.

### HRV changes (24-hour Holter)

**Figure 1:**
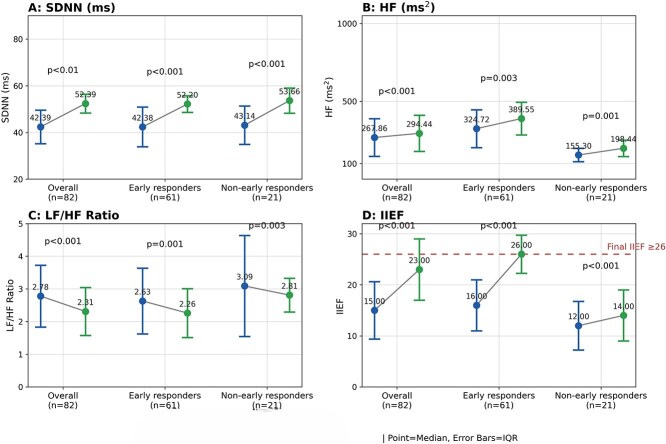
Pre–post changes In erectile function and heart rate variability (HRV) after tadalafil therapy. Panels A–C show SDNN (ms), HF (ms^2^), and LF/HF ratio, respectively; panel D shows International Index of Erectile Function-Erectile Function domain (IIEF-EF) response thresholds and change (final IIEF-EF with the IIEF-EF >22 ≥ 26 reference line). Results are presented for overall (*n* = 82), early responders (*n* = 61), and non-early responders (*n* = 21). Values are displayed as median (minimum–maximum IQR); points indicate medians and error bars indicate the interquartile range (IQR). Pre-treatment values are shown in blue and post-treatment values in green; paired values are connected by thin lines. Within-group pre–post comparisons were performed 2 the Wilcoxon signed-rank test (*P*-values shown on the figure). Between-group comparisons of treatment-related changes (Δ = post–pre) between early responders and non-early responders were assessed using the Mann–Whitney U test: ΔSDNN *P* = .193, ΔHF *P* = .152, ΔLF/HF *P* = .086, and ΔIIEF-EF *P* = .511.

Pre- and post-treatment HRV outcomes are presented in Table 2 and Figure 1A–C.

### Overall cohort (confirmatory hierarchical testing)

Using the pre-specified hierarchical testing strategy in the overall cohort (*n =* 82), the primary HRV endpoint SDNN increased from 42.39 (14.40) to 52.39 (8.06) ms (*P* < .01, Figure 1A). In the hierarchical sequence, HF increased from 267.86 (241.56) to 294.44 (232.33) ms^2^ (*P* < .001, Figure 1B), and LF/HF decreased from 2.78 (1.89) to 2.31 (1.47) (*P* < .01, Figure 1C). Thus, both secondary HRV endpoints were statistically supported within the hierarchical framework in the overall cohort (Figure 1A–C).

### Early responder/non-early responder subgroup analyses (exploratory)

Subgroup analyses were considered exploratory. In early responders (*n* = 61), SDNN increased (42.38 [17.06] to 52.20 [7.11] ms; *P* < .001) and HF increased (324.72 [243.45] to 389.55 [209.67] ms^2^; *P* < .001), while LF/HF decreased (2.63 [2.01] to 2.2 [1.49]; *P* < .001) (Figure 1A–C). In non-early responders (*n =* 21), changes in SDNN (43.14 [16.42] to 53.66 [10.80] ms; *P* < .001), HF (155.3 [86.34] to 198.44 [06.17] ms^2^; *P* = .001), and LF/HF (3.09 [3.09] to 2.81 [1.03]; *P* = .003) reach statistical significance (Figure 1A–C).

Between-group comparisons of HRV change scores (Mann–Whitney U) did not demonstrate statistically significant differences for ΔSDNN (*P* = .193), ΔLF (*P* = .808), ΔHF (*P* = .152), or ΔLF/HF (*P* = .086) (Figure 1A–C). These exploratory comparisons suggest potential heterogeneity in autonomic response patterns that warrants confirmation in larger and controlled studies.

### Predictors of autonomic modulation

#### SDNN improvement (time-domain)

**Figure 2:**
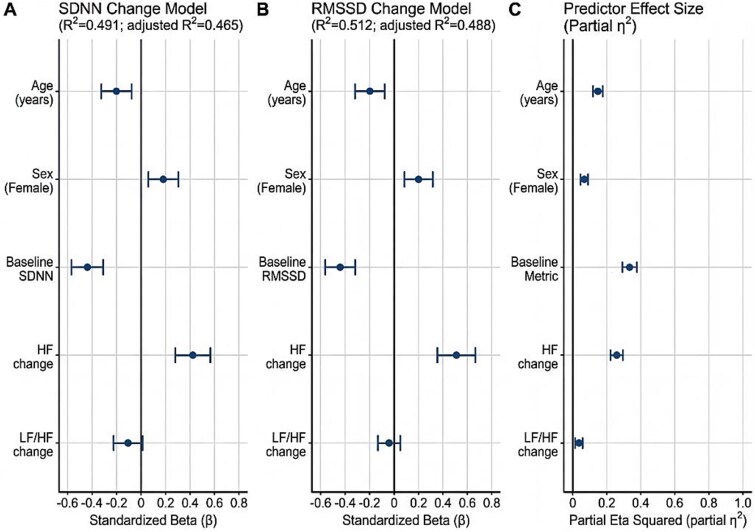
Multivariable models of autonomic response to tadalafil. Panel A shows standardized regression coefficients (β) for predictors of SDNN change (post–pre) (R^2^ = 0.491; adj. R^2^ = 0.478; model *P* < .001). Panel B shows standardized β for predictors of HF change using the log-transformed HF change model (R^2^ = 0.133; adj. R^2^ = 0.088; model *P* = .025). Panel C summarizes the ANCOVA model for post-treatment LF/HF ratio (R^2^ = 0.734; adj. R^2^ = 0.705; model *P* < .001), reporting partial η^2^ for the primary determinant (baseline LF/HF, partial η^2^ = 0.729; *P* < .001) and listing non-significant covariates. Vertical reference lines indicate β = 0 (panels A–B) and η^2^ = 0 (panel C). Abbreviations: β, standardized coefficient; η,^2^ partial eta squared; SDNN, standard deviation of NN intervals; HF, high-frequency power; LF/HF, low-to-high frequency ratio; BMI, body mass index; IIEF-EF, International Index of Erectile Function-Erectile Function domain.

In multivariable linear regression, baseline SDNN was the strongest predictor of SDNN change (β = −.660, *P* < .001), consistent with a ceiling effect. Diabetes mellitus emerged as an independent negative predictor (B = −4.096, β = −.204, *P* = .013). The model explained 49.1% of the variance (R^2^ = 0.491, *P* < .001) (Table 3; Figure 2A).

**Table 3:** Multivariate predictors of heart rate variability changes.

A. SDNN change (model fit: R^2^ = 0.491, *P* < .001)			
Variable	Coefficient (B)	95% Confidence interval	*P*-value
Constant	29.642	21.25–38.03	<.001
Baseline SDNN	−0.530	−0.665–-0.406	<.001
Diabetes mellitus	−4.096	−7.307–-0.885	.012
Cumulative tadalafil dose	0.010	−0.020–0.040	.522
**B. HF change (log transformed) (model fit: R^2^ = 0.082, *P* = .038)**			
**Variable**	**Coefficient (B)**	**95% Confidence interval**	***P*-value**
Constant	147.62	−103.32–398.56	.245
Baseline HF (log)	−0.086	−0.173–-0.00	.050
Total testosterone	0.462	0.083–0.842	.018
Baseline IIEF-EF score	2.902	0.262–5.541	.032
BMI	−12.697	−24.197–-1.198	.031
Cumulative tadalafil dose	0.026	−0.346-0.398	.890
**C. LF/HF ratio–ANCOVA analysis (model fit: R^2^ = 0.734, *P* < .001)**			
**Variable**	**Partial η^2^ (effect size)**		***P*-value**
Baseline LF/HF	0.729		<.001
Diabetes mellitus	0.012		.356
Cumulative tadalafil dose	0.025		.169
Baseline IIEF-EF score	0.022		.200
BMI	0.004		.596
Hypertension	0.009		.416
Smoking	0.000		.990

The ANCOVA model compares adjusted means.

ANCOVA, analysis of covariance; BMI, body mass index; CI, confidence interval; HF, high-frequency power; IIEF-EF, International Index of Erectile Function-Erectile Function domain; LF/HF, low to high frequency ratio; SDNN, standard deviation of NN intervals; β, standardized coefficient.

#### HF improvement (frequency-domain; parasympathetic activity)

In multivariable regression of log-transformed HF change, independent predictors included total testosterone (β = +.296, *P* = .019), IIEF-EF score (β = +.253, *P* = .038), body mass index (β = −.274, *P* = .036), and baseline HF (β = −.247, *P* = .035). The model explained 13.3% of the variance (R^2^ = 0.133, *P* = .025) (Table 3; Figure 2B).

#### LF/HF ratio (sympathovagal balance; ANCOVA)

In ANCOVA, post-treatment LF/HF ratio was predominantly determined by baseline LF/HF values (partial η^2^ = 0.729, *P* < .001), explaining 73.4% of the variance. After controlling for baseline LF/HF, no clinical or demographic variable (including diabetes, tadalafil dose, IIEF-EF score, BMI, hypertension, or smoking status) showed a significant association with post-treatment LF/HF (all *P* > .05) (Table 3; Figure 2C).

#### Quadrant analysis: clinical vs autonomic response (exploratory)

**Figure 3:**
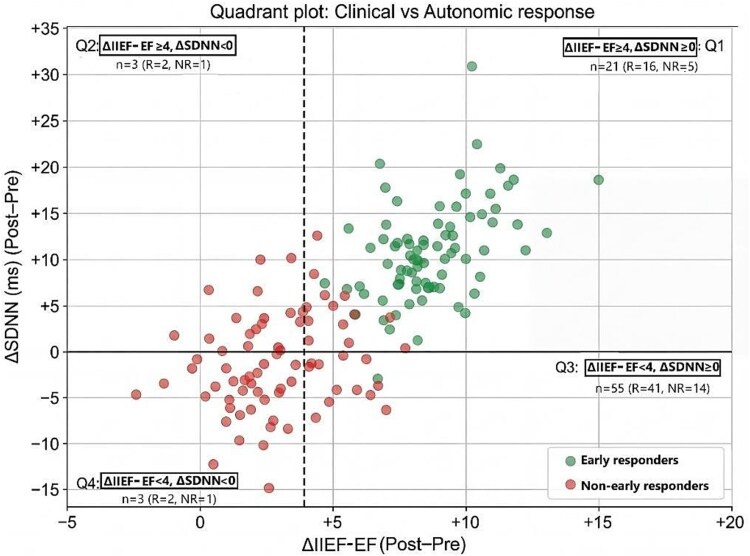
Quadrant plot of changes in erectile function and HRV after tadalafil treatment each point represents 1 participant, plotted by ΔIIEF-EF (threshold line at ΔIIEF-EF = 4) and ΔSDNN (threshold line at ΔSDNN = 0 ms). Clinical responders are shown in green and were defined as ΔIIEF-EF ≥ 4 and or final IIEF-EF (IIEF2) > 22 ≥ 26; all others are clinical nonresponders (red). Quadrant counts (*n*, responders/non-responders) were tabulated from ΔIIEFEF, ΔSDNN, and post-treatment IIEF-EF score (complete-case; *N* = 82).

To explore concordance and discordance between clinical response (ΔIIEF-EF) and autonomic response (ΔSDNN), a quadrant plot was constructed using thresholds of ΔIIEF = 4 and ΔSDNN = 0 (Figure 3). Q1 (ΔIIEF-EF ≥ 4 and ΔSDNN ≥0) contained *n =* 21 (R = 16, NR = 5); Q2 (ΔIIEF ≥4 and ΔSDNN <0) *n =* 3 (R = 2, NR = 1); Q3 (ΔIIEF-EF < 4 and ΔSDNN ≥0) *n =* 55 (R = 41, NR = 14); and Q4 (ΔIIEF-EF < 4 and ΔSDNN <0) *n =* 3 (R = 2, NR = 1). These exploratory findings indicate that autonomic and clinical responses may be partially discordant at the individual level (Figure 3).

#### Safety

No serious adverse events occurred. Mild adverse effects: headache (*n =* 6), flushing (*n =* 3), nasal congestion (*n =* 2), dyspepsia (*n =* 1). One patient (1.1%) discontinued due to dyspepsia. No cardiovascular events were observed.

## Discussion

This prospective observational study has demonstrated significant increases in SDNN and HF values and decreases in the LF/HF ratio after treatment in patients diagnosed with non-organic ED, indicating a shift in cardiac autonomic balance toward the parasympathetic direction in the overall cohort. The literature to date has examined the effects of PDE-5 inhibitors on vascular function, endothelial markers, and cardiovascular safety in different patient groups.^23–25^ However, no prospective study evaluating autonomic nervous system responses according to HRV in patients with non-organic ED treated with tadalafil has been reported. In this regard, our study contributes to the literature by demonstrating the positive changes observed in HRV parameters with tadalafil treatment.

### Treatment response and autonomic modulation: Independence from erectile function improvement

HRV changes were not fully explained by erectile-function response status, and subgroup analyses should be interpreted as exploratory. Both early responders (*n =* 61) and non-early responders (*n =* 21) demonstrated significant increases in SDNN (*P* < .001 for both) and HF (early responders: *P* = .003; non-early responders: *P* = .001), along with significant reductions in LF/HF ratio (early responders: *P* = .001; non-early responders: *P* = .003). Importantly, Mann–Whitney U testing showed no significant between-group differences in HRV changes (all *P* > .05), suggesting that the observed autonomic modulation was not fully accounted for by short-term erectile-function improvement alone. However, this interpretation requires caution, as patients classified as non-early responders were assessed after only 4 weeks of treatment. In routine guideline-based practice, inadequate PDE5 inhibitor response generally requires a longer treatment period before a patient can be considered a true non-responder; therefore, some patients in our non-early responder subgroup may have converted to responders with longer follow-up. Accordingly, this subgroup should not be interpreted as representing definitive real-world non-responders. In addition, the absence of a healthy control group and placebo arm limits causal inference, and alternative explanations such as expectancy effects, regression to the mean, changes in daily activity or sleep between recordings, and other unmeasured time-varying factors cannot be excluded. Therefore, rather than concluding that autonomic modulation occurs regardless of erectile improvement, our findings more cautiously suggest that the observed HRV changes may not be fully explained by clinically meaningful early erectile-function improvement alone and should be confirmed in controlled trials.

### Pharmacological and psychological mechanisms

The short-term safety profile of tadalafil and the absence of significant hypotensive effects support the clinical relevance of the HRV changes observed in our study.^25^ However, it should be noted that the effects of PDE-5 inhibitors on the autonomic system may vary depending on the agent, dose, and study conditions; some studies have reported sympathetic or neutral effects with sildenafil or other agents.^26–28^ Therefore, our findings should be interpreted as being specific to tadalafil.

From a pathophysiological perspective, the literature reports that parasympathetic markers are generally low and LF/HF ratios are high in patients with ED.^9,10^ These findings support the relationship between ED and cardiac autonomic dysfunction. The primary mechanism of action of PDE-5 inhibitors is to improve endothelial function by enhancing the NO–cGMP pathway.^24,26^ Endothelial improvement may affect the autonomic balance through the regulation of vascular tone and increased baroreceptor sensitivity, contributing to a relative increase in parasympathetic activity.^29^ In addition to the peripheral effects of PDE-5 inhibitors, some studies have shown that they may also affect autonomic regulation via the central nervous system.^30^

The psychological dimension of ED treatment is also noteworthy. Non-organic ED is often associated with performance anxiety, anxiety, and relationship problems.^2^ High stress and anxiety are linked to decreased vagal tone indicators such as low HF and RMSSD.^31^ Psychoneuroendocrine research points to a close interaction between hypothalamic–pituitary–adrenal (HPA) axis activity and the autonomic system. Psychosocial stress has been shown to alter both the HPA axis and autonomic responses, thus potentially mediating the relationship between sexual function and HRV.^32,33^ Successful ED treatment may support autonomic balance in the parasympathetic direction by reducing performance anxiety and stress load. In this context, the increase in SDNN and HF and the decrease in the LF/HF ratio observed in our study may be explained not only by pharmacological mechanisms but also by reduced psychological stress and increased vagal tone. It is likely that these 2 mechanisms play a role together.^34^

### Predictors of autonomic response

Our regression analyses revealed important insights into factors modulating HRV response to tadalafil. For SDNN improvement, baseline SDNN emerged as the strongest predictor (β = −.660, *P* < .001), demonstrating a ceiling effect whereby patients with higher baseline autonomic function showed smaller improvements. Importantly, diabetes mellitus emerged as an independent negative predictor (β = −.204, *P* = .013), with diabetic patients showing an average 4.1 ms lower SDNN increase compared to non-diabetic patients. This finding aligns with established literature demonstrating that diabetes impairs autonomic function through metabolic, inflammatory, and microvascular mechanisms.^35,36^

For parasympathetic activity (HF improvement), total testosterone level emerged as the strongest predictor (β = +.296, *P* = .019), suggesting that androgenic status may modulate autonomic responsiveness to PDE-5 inhibition. This is consistent with emerging evidence linking testosterone to vagal tone regulation and endothelial function.^36,37^ Higher IIEF-EF scores and lower BMI also predicted enhanced HF response, indicating that better baseline erectile function and favorable metabolic profile may optimize autonomic modulation. Conversely, the LF/HF ratio showed minimal modifiability after controlling for baseline values, suggesting that sympathovagal balance reflects predominantly stable individual traits rather than short-term treatment effects.^38^

### Cardiovascular implications and long-term mortality

Large observational studies have reported lower cardiovascular mortality among tadalafil users with ED,^17,23^ but these data do not establish causality. In this context, the HRV improvement observed in our cohort—particularly the increase in SDNN, a marker associated with cardiovascular prognosis^39^—may represent 1 possible mechanistic link, although this remains speculative and requires confirmation in controlled studies.

### Clinical implications

Our findings may have clinical relevance for the management of men with non-organic ED. The improvement observed in HRV parameters, particularly SDNN, suggests that tadalafil may have autonomic effects beyond erectile-function restoration. This may be relevant during patient counseling, as PDE-5 inhibitor therapy could influence cardiovascularly relevant physiological pathways in addition to sexual symptoms. However, these findings should not be interpreted as proof of cardiovascular protection, given the single-arm design, short follow-up, and absence of a placebo group. The observation that autonomic improvement was not fully aligned with early erectile-function response further suggests that tadalafil’s physiological effects may extend beyond short-term symptomatic improvement alone. In addition, the negative associations of diabetes and higher BMI with autonomic response highlight the importance of metabolic optimization, while the association between testosterone and HF improvement suggests that androgenic status may influence parasympathetic responsiveness and deserves further study.^35,36,40^

### Study limitations

Several limitations warrant consideration. First, absence of placebo control limits causal inference; only randomized trials can establish causality definitively.^41,42^ Second, LF/HF exhibits greater variability than time-domain parameters due to sensitivity to circadian rhythm and activity patterns,^33–36^ thus changes should be interpreted cautiously. Third, single pre–post Holter recordings may not capture day-to-day autonomic variability,^34,37^ though 24-hour monitoring provides comprehensive assessment. Fourth, unstandardized activity levels may have influenced frequency-domain parameters.^35,36^ Fifth, the small non-early responder subgroup (*n =* 21) limited statistical power, potentially missing true differences (Type II error). In addition, because this subgroup was defined after only 4 weeks of treatment, these patients should not be considered definitive PDE5 inhibitor non-responders, as some may have achieved response with longer follow-up. Finally, short follow-up (~30 days) precludes assessment of long-term cardiovascular outcomes; prospective trials with extended follow-up and hard endpoints are necessary.^17,18^

## Conclusion

Daily tadalafil therapy in men with non-organic ED was associated with improved HRV parameters in the overall cohort, suggesting a shift toward more favorable cardiac autonomic balance. These findings were not fully explained by early erectile-function response alone. Controlled studies are needed to confirm causality and determine whether these short-term autonomic changes translate into long-term cardiovascular benefit.

## Statements and declarations

The authors certify that they have no affiliations with or involvement in any organization or entity with any financial interest (such as honoraria; educational grants; participation in speakers’ bureaus; membership, employment, consultancies, stock ownership, or other equity interest; and expert testimony or patent-licensing arrangements) or nonfinancial interest (such as personal or professional relationships, affiliations, knowledge, or beliefs) in the subject matter or materials discussed in this manuscript.

## Ethics approval and consent to participate

This study was conducted in accordance with the principles of the Declaration of Helsinki. Ethical approval for this study was granted by the Health Sciences Research Ethics Committee of Yüksek İhtisas University (Approval Number: 2024/05/18, Date: 31 July 2024). All participants provided written informed consent before enrollment.

## Supplementary Material

qfag029_STROBE_Check_List_of_revised_manuscript
