# Supplementary material for: Daily tadalafil administration improves cardiac autonomic regulation in men with non-organic erectile dysfunction: a prospective heart rate variability study
Source: Sex Med. 2026 May 11;14(4):qfag029. doi: 10.1093/sexmed/qfag029 (PMC13158229; doi:10.1093/sexmed/qfag029)
Supplement: qfag029_STROBE_Check_List_of_revised_manuscript [file qfag029_strobe_check_list_of_revised_manuscript.docx]

# STROBE STATEMENT — CHECKLIST OF ITEMS INCLUDED IN REPORTS OF COHORT STUDIES

Daily Tadalafil Administration Improves Cardiac Autonomic Regulation in Men with Non-Organic Erectile Dysfunction: A Prospective Heart Rate Variability Study

**Manuscript ID:** SM-26-0325

**Study Design:** Prospective observational cohort study

**Submission Type:** Revised Manuscript (Supplementary File)

| **No.** | **Recommendation** | **Page No.** | **Comment / Where Reported** |
| --- | --- | --- | --- |
| **TITLE AND ABSTRACT** | | | |
| 1 | (a) Indicate the study’s design with a commonly used term in the title or the abstract | **1** | Title includes "Prospective Heart Rate Variability Study". Abstract explicitly states "Prospective, single-arm observational cohort study". |
| 1 | (b) Provide in the abstract an informative and balanced summary of what was done and what was found | **1-2** | Abstract summarizes background, aim, methods (n=82, tadalafil 5mg, Holter), results (SDNN p=0.021, HF p=0.015), and conclusion. |
| **INTRODUCTION** | | | |
| 2 | Explain the scientific background and rationale for the investigation being reported | **3** | Introduction details link between ED and CVD risk, autonomic dysfunction mechanisms, and lack of data on tadalafil's direct HRV effects. |
| 3 | State specific objectives, including any prespecified hypotheses | **3-4** | Introduction ends with specific hypothesis: "Does 30-day daily tadalafil administration significantly improve cardiac autonomic regulation... and does the magnitude differ between early responders and non-early responders?" |
| **METHODS** | | | |
| 4 | Present key elements of study design early in the paper | **4** | Materials & Methods: "Prospective, single-arm observational study conducted between August 15, 2024, and February 15,  2025." |
| 5 | Describe the setting, locations, and relevant dates, including periods of recruitment, exposure, follow-up, and data collection | **4** | Setting: Outpatient Clinic of Yüksek İhtisas University. Dates: August 15, 2024 – February 15, 2025. |
| 6 | (a) Give the eligibility criteria, and the sources and methods of selection of participants. Describe methods of follow-up | **4-5** | Eligibility: Men with non-organic ED. Exclusion: CVD, antiarrhythmics. Selection: Consecutive sampling. Follow-up: ~30 days (29.6 ± 2.9 days). |

| **No.** | **Recommendation** | **Page No.** | **Comment / Where Reported** |
| --- | --- | --- | --- |
| 6 | (b) For matched studies, give matching criteria and number of exposed and unexposed | **N/A** | Not applicable. Single-arm cohort study without matching. |
| 7 | Clearly define all outcomes, exposures, predictors, potential confounders, and effect modifiers. Give diagnostic criteria, if applicable | **5-6** | Outcomes: ΔSDNN (primary), ΔHF, ΔLF/HF (secondary). Exposure: Tadalafil 5-10mg. Predictors: Age, BMI, Diabetes, Testosterone. Diagnosis: AUA guideline for non-organic ED. |
| 8 | For each variable of interest, give sources of data and details of methods of assessment (measurement). Describe comparability of assessment methods if there is more than one group | **5-6** | Data sources: 24-hr Holter (BTL-08, CardioPoint software). Assessment: Task Force standards for HRV. IIEF-EF questionnaire. Same methods used pre/post for all patients. |
| 9 | Describe any efforts to address potential sources of bias | **4, 6** | Selection bias: Consecutive sampling. Detection bias: Blinded assessment (cardiologists blinded to IIEF, urologists to HRV). Measurement bias: Standardized 08:00-08:30 start time. |
| 10 | Explain how the study size was arrived at | **7** | Sample size based on available consecutive patients within study period. Limitations section acknowledges small non-early responder subgroup (n=21). |
| 11 | Explain how quantitative variables were handled in the analyses. If applicable, describe which groupings were chosen and why | **7** | Continuous variables: Mean ± SD (normal) or Median (min-max) (IQR) (non-normal). Groupings: Early responders vs. Non-early responders based on MCID or final IIEF ≥26. |
| 12 | (a) Describe all statistical methods, including those used to control for confounding | **7-8** | Wilcoxon signed-rank (within-group), Mann–Whitney U (between-group). Multivariable linear regression & ANCOVA used to control for baseline confounders. |
| 12 | (b) Describe any methods used to examine subgroups and interactions | **8** | Subgroup analysis performed for Early Responders (n=61) vs. Non-Early Responders (n=21). Interaction terms checked in regression models. |
| 12 | (c) Explain how missing data were addressed | **8** | Complete-case analysis (n=82). No imputation performed for main analysis. |
| 12 | (d) If applicable, explain how loss to follow-up was addressed | **8** | Loss to follow-up reported (n=6). Reasons provided (withdrawal, lost contact, side effects). |
| 12 | (e) Describe any sensitivity analyses | **8** | Sensitivity analysis performed assuming worst-case scenario for dropouts (n=88); results remained robust (p=0.027 vs p=0.021 for SDNN). |
| **RESULTS** | | | |
| 13 | (a) Report numbers of individuals at each stage of study—eg numbers potentially eligible, examined for eligibility, confirmed eligible, included in the study, completing follow-up, and analysed | **7** | Screened: 122. Excluded: 34. Enrolled: 88. Lost to follow-up: 6.  Analyzed: 82 (Figure 1: Participant Flow Diagram) |
| 13 | (b) Give reasons for non-participation at each stage | **7** | Exclusions: 26 (CVD/antiarrhythmics), 8 (poor Holter quality). Dropouts: 3 withdrew, 2 lost contact, 1 dyspepsia. |
| 13 | (c) Consider use of a flow diagram | **7** | Figure 1 (Flowchart) is referenced in the text to illustrate participant flow. |

| **No.** | **Recommendation** | **Page No.** | **Comment / Where Reported** |
| --- | --- | --- | --- |
| 14 | (a) Give characteristics of study participants (eg demographic, clinical, social) and information on exposures and potential confounders | **Table 1** | Table 1 reports age (46.8±5.0), BMI, comorbidities (diabetes, hypertension), smoking status, and baseline labs. |
| 14 | (b) Indicate number of participants with missing data for each variable of interest | **8** | Complete data available for n=82 analyzed participants. |
| 14 | (c) Summarise follow-up time (eg, average and total amount) | **7** | Mean follow-up duration: 29.6 ± 2.9 days. |
| 15 | Report numbers of outcome events or summary measures over time | **Table 2** | Table 2 reports pre- and post-treatment values for HRV parameters and IIEF-EF scores for overall cohort and subgroups. |
| 16 | (a) Give unadjusted estimates and, if applicable, confounder-adjusted estimates and their precision (eg, 95% confidence interval). Make clear which confounders were adjusted for and why they were included | **9-10** | Unadjusted: Wilcoxon results (p-values). Adjusted: Multivariable regression (Table 3) reports β coefficients, 95% CI, and p-values controlling for age, BMI, diabetes, etc. |
| 16 | (b) Report category boundaries when continuous variables were categorized | **7** | Response categorization boundaries: Severity-adjusted MCID (≥2/5/7 pts) OR Final IIEF ≥26. |
| 16 | (c) If relevant, consider translating estimates of relative risk into absolute risk for a meaningful time period | **N/A** | Not applicable. Study reports physiological parameters (HRV), not clinical event risks. |
| 17 | Report other analyses done—eg analyses of subgroups and interactions, and sensitivity analyses | **10-11** | Subgroup analysis (Early vs Non-early responders). Hierarchical testing strategy (SDNN → HF → LF/HF). Quadrant analysis of clinical vs autonomic response. |
| **DISCUSSION** | | | |
| 18 | Summarise key results with reference to study objectives | **11** | Discussion begins by stating tadalafil improved autonomic function (increased SDNN/HF, decreased LF/HF) independent of erectile response, meeting primary objective. |
| 19 | Discuss limitations of the study, taking into account sources of potential bias or imprecision. Discuss both direction and magnitude of any potential bias | **14-15** | Limitations section discusses lack of placebo (causality), short follow-up (30 days), small non-responder sample (n=21), and open-label design (bias). |
| 20 | Give a cautious overall interpretation of results considering objectives, limitations, multiplicity of analyses, results from similar studies, and other relevant evidence | **12-14** | Results interpreted as "beneficial modulation" rather than definitive mortality reduction. Comparison made with previous studies on PDE5Is and HRV. |
| 21 | Discuss the generalisability (external validity) of the study results | **15** | Findings generalized to men with non-organic ED; caution advised for other populations. Suggests potential relevance for cardiovascular risk management. |
| **OTHER INFORMATION** | | | |
| 22 | Give the source of funding and the role of the funders for the present study and, if applicable, for the original study on which the present article is based | **15** | Stated as: "No funding was received for this study." |
